# Supplementary material for: Prognostic role and biologic features of Musashi-2 expression in colon polyps and during colorectal cancer progression
Source: PLoS One. 2021 Jul 8;16(7):e0252132. doi: 10.1371/journal.pone.0252132 (PMC8266110; doi:10.1371/journal.pone.0252132)
Supplement: S1 Table — For human cell lines, the lentiviral vectors Tet-pLKO-puro (Addgene, Plasmid #21915) was used for inducible expression of shRNAs. (DOCX) [file pone.0252132.s001.docx]

| Name | Vector | Type of insert | Type of expression | Cell line origin |
| --- | --- | --- | --- | --- |
| RKO | Tet-pLKO-puro | Empty | no | Human |
| RKO sh1 | Tet-pLKO-sh1-puro | MSI2-shRNA1 | Inducible | Human |
| RKO sh2 | Tet-pLKO-sh2-puro | MSI2-shRNA2 | Inducible | Human |
| HCT-116 | Tet-pLKO-puro | Empty | no | Human |
| HCT-116 sh1 | Tet-pLKO-sh1-puro | MSI2-shRNA1 | Inducible | Human |
| HCT-116 sh2 | Tet-pLKO-sh2-puro | MSI2-shRNA2 | Inducible | Human |

**Supplementary table S1,** List of cell line derivatives used in the study. For human cell lines, the lentiviral vectors Tet-pLKO-puro (Addgene, Plasmid #21915) was used for inducible expression of shRNAs.
