# Supplementary material for: Prognostic role and biologic features of Musashi-2 expression in colon polyps and during colorectal cancer progression
Source: PLoS One. 2021 Jul 8;16(7):e0252132. doi: 10.1371/journal.pone.0252132 (PMC8266110; doi:10.1371/journal.pone.0252132)
Supplement: S2 Table — Table lists of single stranded DNA (ssDNA) oligos used for generation of Tet-pLKO-puro vectors expressing specific shRNAs, which used for lentiviral infection and selection of stable cell lines. MSI2 targeting sequences are underscored. (DOCX) [file pone.0252132.s002.docx]

|  | ssDNA oligos |
| --- | --- |
| Human, sh1 | Fw: 5`- CCGGGTGGAAGATGTAAAGCAATATCTCGAGATATTGCTTTACATCTTCCACTTTTT |
|  | Rev: 5`-AATTAAAAAGTGGAAGATGTAAAGCAATATCTCGAGATATTGCTTTACATCTTCCAC |
| Human, sh2 | Fw: 5`-CCGGCCCAACTTCGTGGCGACCTATCTCGAGATAGGTCGCCACGAAGTTGGGTTTTT |
|  | Rev: 5`-AATTAAAAACCCAACTTCGTGGCGACCTATCTCGAGATAGGTCGCCACGAAGTTGGG |

**Supplementary table S2,** **DNA oligonucleotides used for construction of shRNA vectors.** Table lists of single stranded DNA (ssDNA) oligos used for generation of Tet-pLKO-puro vectors expressing specific shRNAs, which used for lentiviral infection and selection of stable cell lines. MSI2 targeting sequences are underscored.
