# Supplementary material for: Prognostic role and biologic features of Musashi-2 expression in colon polyps and during colorectal cancer progression
Source: PLoS One. 2021 Jul 8;16(7):e0252132. doi: 10.1371/journal.pone.0252132 (PMC8266110; doi:10.1371/journal.pone.0252132)
Supplement: S4 Table — List of primers used for SYBR Green assay, and TaqMan gene expression assay, used for qRT-PCR analysis of gene expression. (DOCX) [file pone.0252132.s004.docx]

| Gene symbol (H)-human; | SYBR Green | Taqman Life Technologies |
| --- | --- | --- |
| *MSI2* (H) | Fw: 5`-AACTACCAACAGGCACAGAG  Rev: 5`-CTGAGCTTTCTTACATTCTACCATT |  |
| *18S rRNA* (H) |  | Fw: 5` GCTCTTTCTCGATTCCGT  Rev: 5`- CCAGAGTCTCGTTCGTTATC  Probe: 6fam- TTCTTAGTTGGTGGAGCGATTTGT- Iowa blackFQ |
| CDH1 (H) | Fw: 5`-AGCGTGTGTGACTGTGAA  Rev: 5`-CGAAGAAACAGCAAGAGCAG |  |
| TGFβ-1 | Fw: 5`-CCGACTACTACGCCAAGGA  Rev: 5`-GTTCAGGTACCGCTTCTCG |  |
| ZO-1 | Fw: 5`-CAACAGCATCCTTCCACCTT  Rev: 5`-GTAAGCGCAGCTCCACAG |  |

**Supplementary table S4, Primers used for RT-PCR to quantify gene expression.** List of primers used for SYBR Green assay, and TaqMan gene expression assay, used for qRT-PCR analysis of gene expression.
