# Supplementary material for: Prognostic role and biologic features of Musashi-2 expression in colon polyps and during colorectal cancer progression
Source: PLoS One. 2021 Jul 8;16(7):e0252132. doi: 10.1371/journal.pone.0252132 (PMC8266110; doi:10.1371/journal.pone.0252132)
Supplement: S1 File — (PDF) [file pone.0252132.s005.pdf]

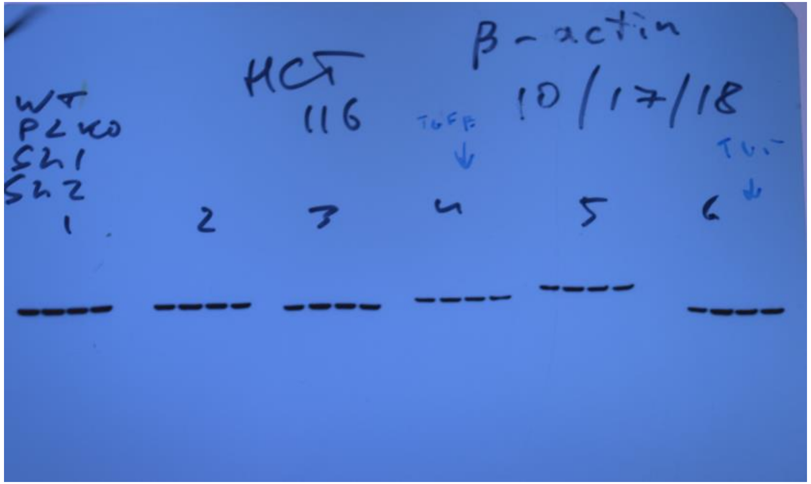
β-actin
HCT 116
10/17/18
WT
PLKO
Sh1
Sh2
TGFβ
1
2
3
4
5
6

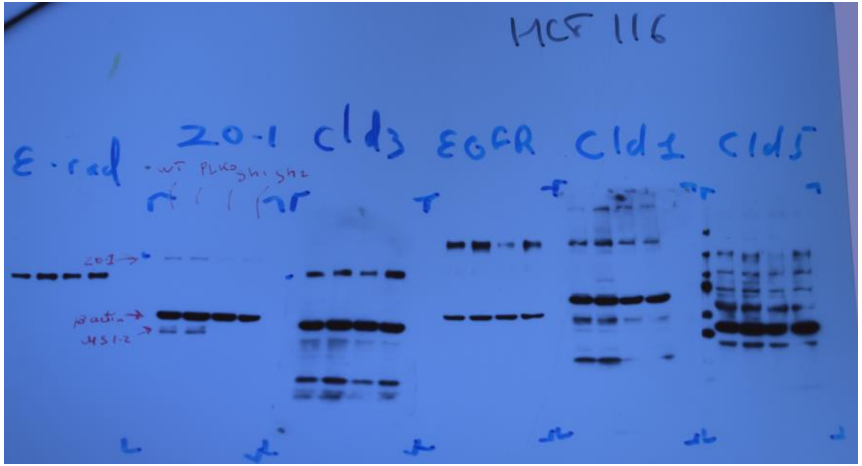
HCT 116
E-cad
ZO-1
Cld3
EGFR
Cld1
Cld5
WT PLKO sh1 sh2
ZO-1
β actin
MSI-2

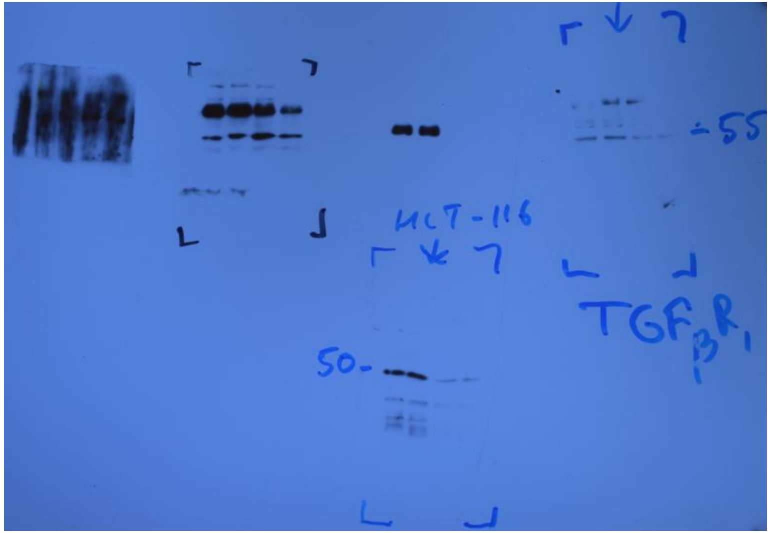
55
HCT-116
TGFβR1
50

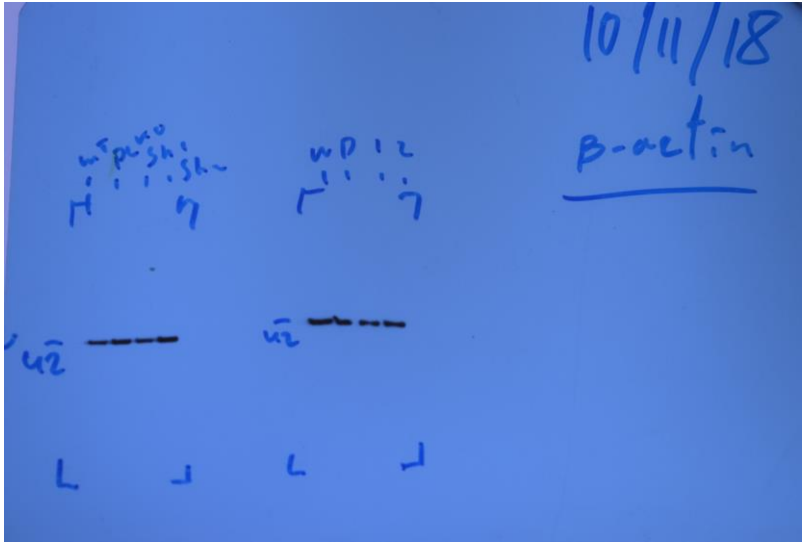
10/11/18
β-actin
WT PLKO Sh1 Sh2
WP 1 2

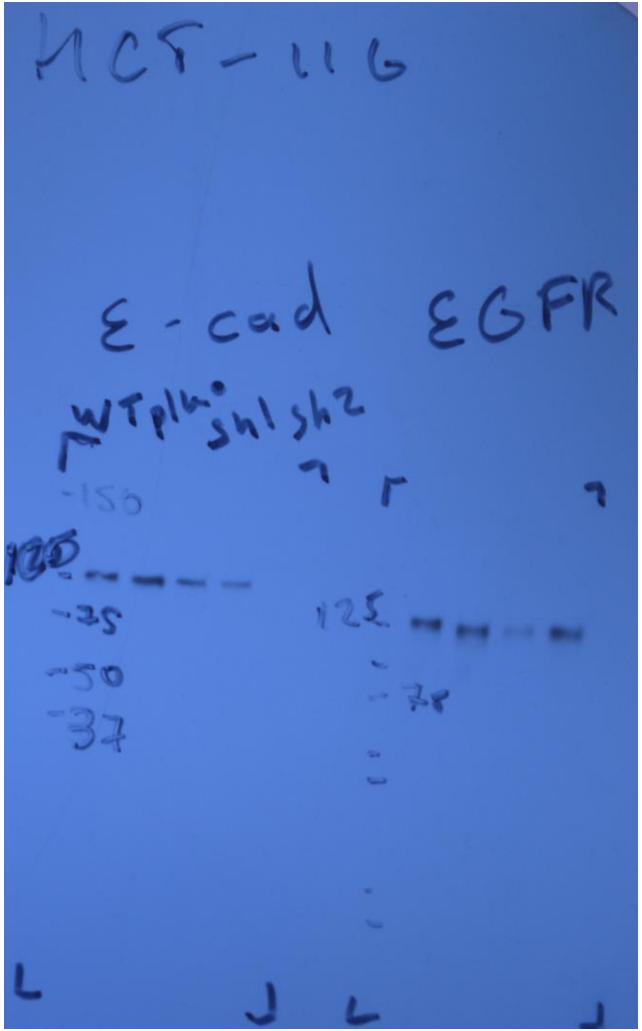
HCT-116
E-cad
EGFR
WT plko sh1 sh2
150
100
75
50
37
125

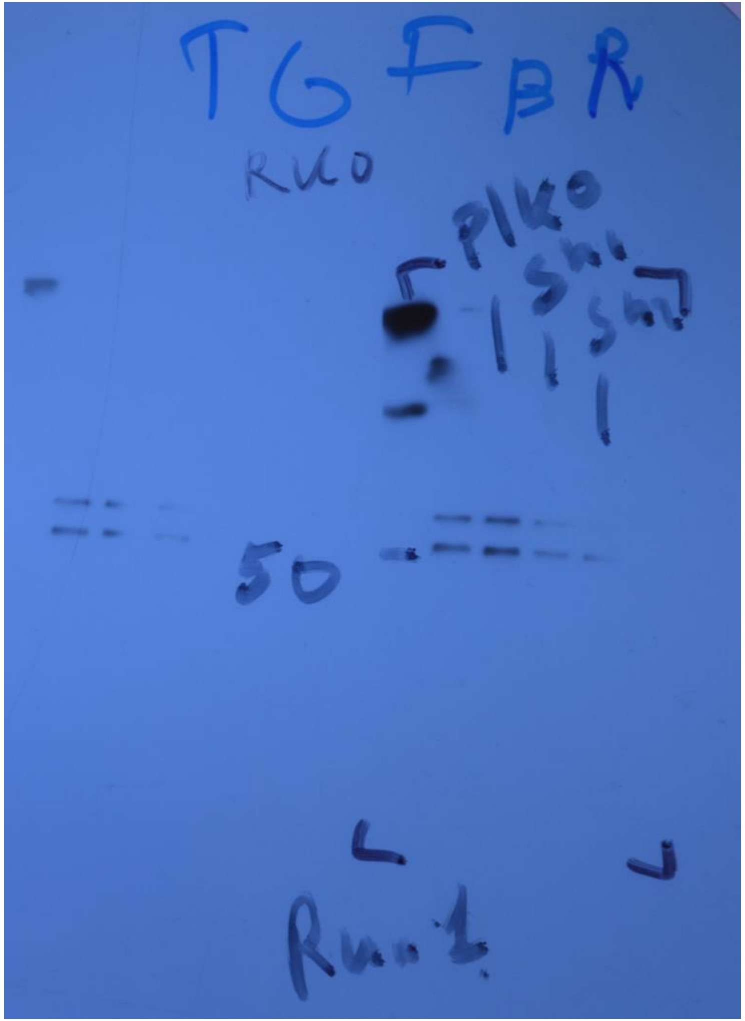
TGFBR
RKO
plko
sh1
sh2
50
Run 1

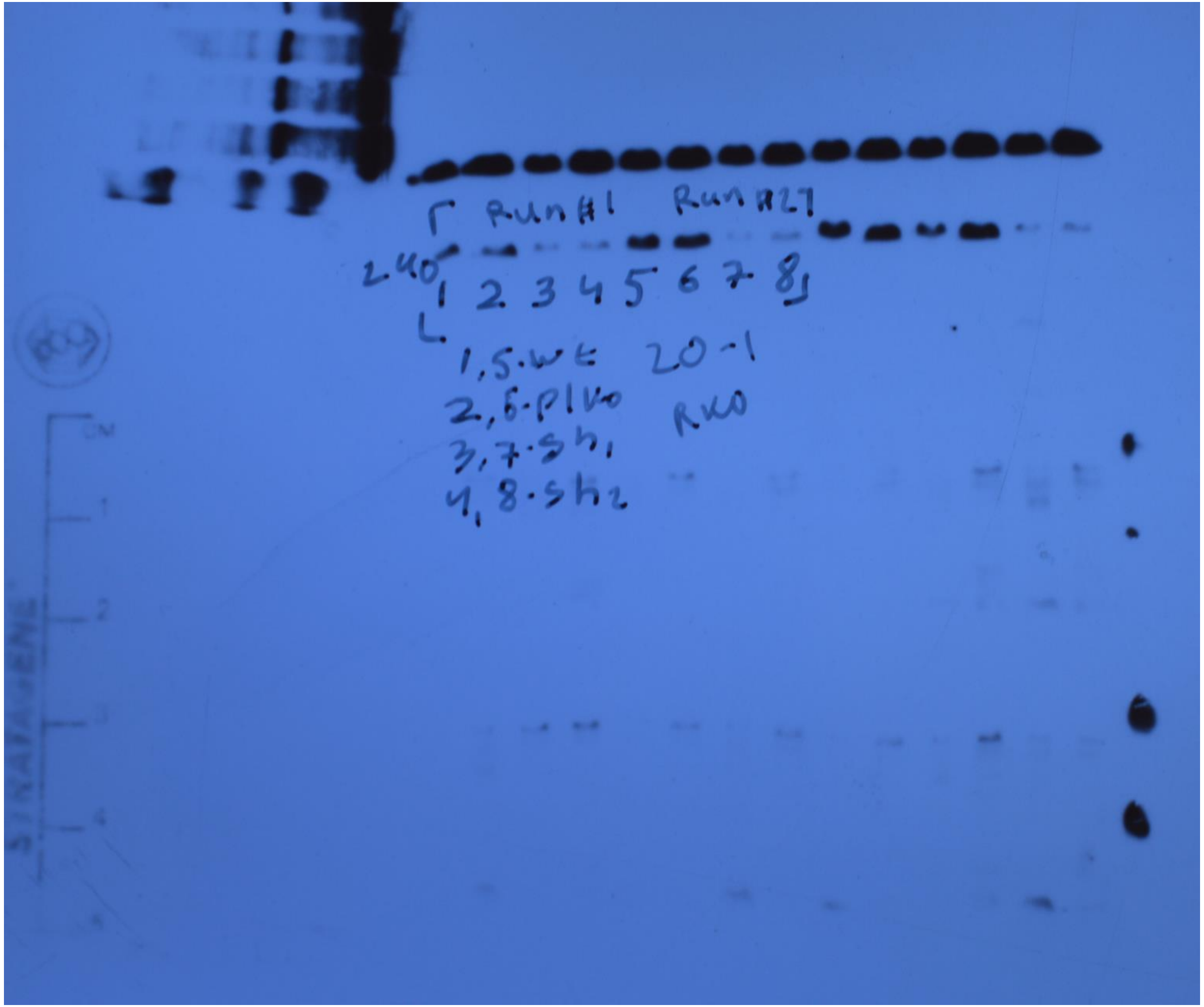
Run #1
Run #2
240
1 2 3 4 5 6 7 8
1,5 wt
20-1
2,6 p1ko
RKO
3,7 sh1
4,8 sh2

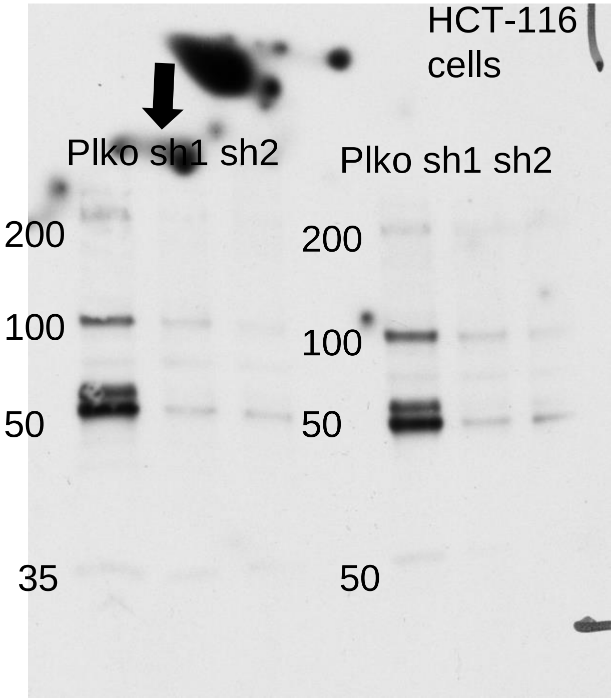
HCT-116
cells
Plko sh1 sh2
Plko sh1 sh2
200
100
50
35
200
100
50
50

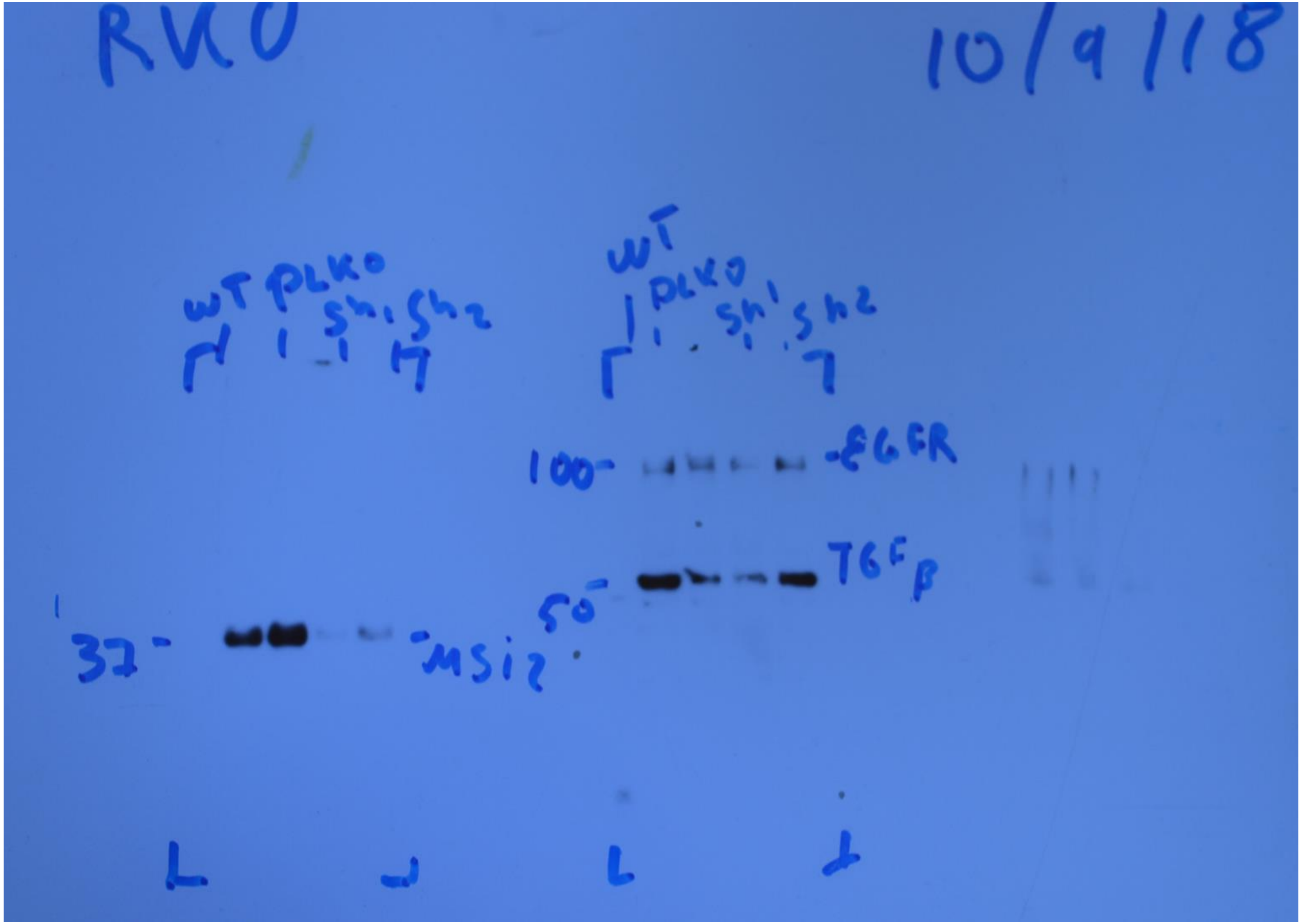
RKO
10/9/18
WT pLKO sh1 sh2
WT
pLKO sh1 sh2
100-
-EGFR
TGFβ
50-
37-
MSI2

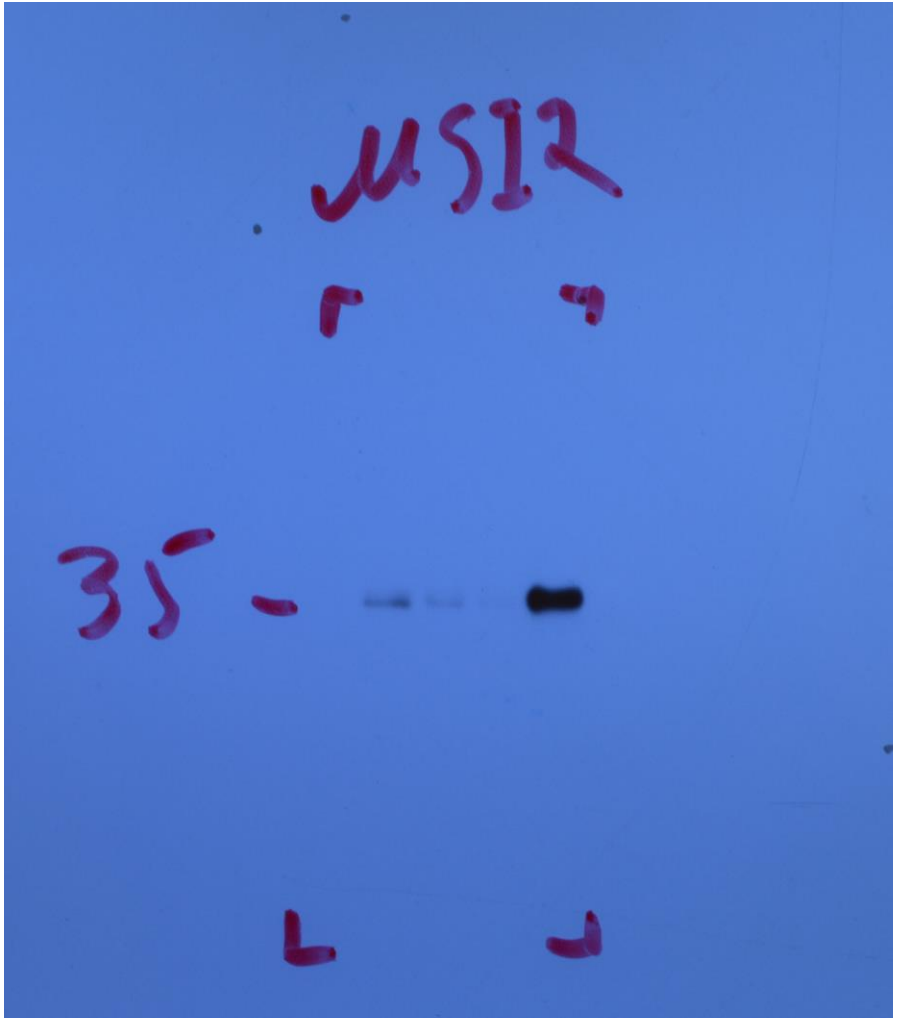
MSI2
35

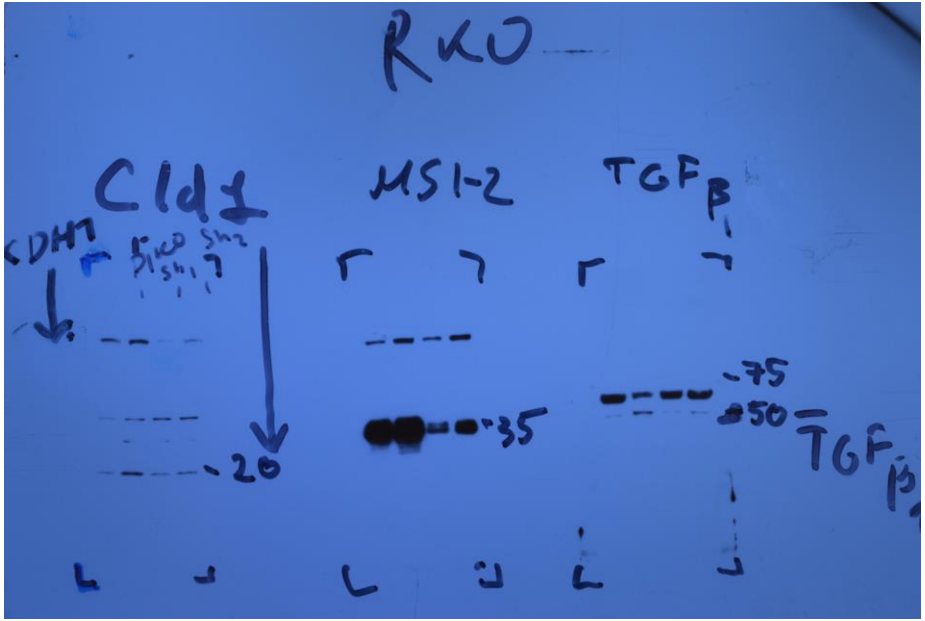
RKO
Cld1
MSI-2
TGFβ
CDH1
RKO
Sh1
Sh2
20
35
75
50
TGFβ
