# Supplementary material for: Prognostic role and biologic features of Musashi-2 expression in colon polyps and during colorectal cancer progression
Source: PLoS One. 2021 Jul 8;16(7):e0252132. doi: 10.1371/journal.pone.0252132 (PMC8266110; doi:10.1371/journal.pone.0252132)
Supplement: Attachment — Submitted filename: Response to Reviewers.docx [file pone.0252132.s006.docx]

December 9, 2020

Punita Dhawan, PhD

Editorial Office

*PLoS One*

Dear Dr. Dhawan,

We here submit our revised original research article, “**“"Prognostic role and biologic features of Musashi-2 expression in colon polyps and during colorectal cancer progression"”** (PONE-D-20-17649), to be considered for publication in *PLoS One*. We greatly appreciate the expert comments and suggestions from the reviewers. We have addressed most of the points raised by the reviewers, and the appropriate changes have been made to the manuscript, as detailed below. A small number of points could not be addressed due to technical reasons which we explain in the following point-by-point response letter and additional data attached. We hope that you agree that the revised manuscript is significantly improved and is now acceptable for publication.

**Reviewer #1:** RNA-binding protein Musashi-2 (MSI2) controls the translation of proteins that support stem cell identity and lineage determination and is associated with progression in some cancers. The present study explore the prognostic significance of MSI2 in CRC polyps and colon cancer. It is a well-planned study that explicitly signifies the role of MSI2 in CRC.

Response: We thank the Reviewer for the positive assessment of the study.

**Point #1**. …The data on its role in initiation is still unclear. In order to clarify its role and prognostic significance, it will be interesting to see MSI2 expression alteration in hyperplastic, and serrated polyp types.

**Response #1**: We have added new images showing the expression of MSI2 in 1 hyperplastic polyp and 1 serrated adenoma (revised Figure 1). These data suggest that MSI2 is not expressed in these early stage lesions. However, the low number of samples available does not allow us to draw statistically significant conclusions, so we are cautious in discussing these results.

**Point #2**. How microsatellite instability impact MSI2 expression and does its differential expression is associated with mutational status of tumor.

**Response #2**: This is an important point. In our study, we analyzed 39 tumors that were microsatellite stable, and 5 tumors that had microsatellite instability (numbers now indicated in revised Table 1). We found no impact of microsatellite instability status on MSI2 expression level (p=0.932). We have added a sentence to the results to make this point.

**Minor Suggestion**: A high level of MSI2 expression in the metastasis was also associated with lymph node positivity (median values with quartiles 270 [250;280] for N0 versus 265 [242;280] for N1 versus 280 [275;290] for N2; average values, 268 versus 262 versus 281; p=0.04), and with higher differentiation grade of the primary tumor (p = 0.000). Clearly mention the p-value observed for the study.

**Response for suggestion**: We have clarified the description of the results with associated p-values in the main text of the manuscript.

**Reviewer #2:** The manuscript entitled "Prognostic role and biologic features of Musashi-2 expression in colon polyps and during colorectal cancer progression" by Leonid Kharin et al. Here authors, addressed the critical role of Musashi-2 (MSI2) in colorectal cancer and metastasis. The idea of this article is exciting. The author showed a stage-wise increase in the expression of MSI2. Also, it showed the critical role of MSI cell proliferation and growth. However, colon cancer-promoting role of MSI2 is already reported. The techniques or methodology used is up to date. However, the following points need to be addressed.

Response: We thank the Reviewer for the positive assessment of the study.

**Point #1**. In one section of the results, the author stated that the expression of MSI2 in primary tumors did not correlate with CDH1 expression. However, in another section using the TCGA data set, the author showed a positive correlation between MSI2 protein expression and CDH1 protein. This ambiguity needs to be addressed.

**Response #1**: We apologize for the apparent contradiction of these data. Due to limited tissue for both MSI2 and CDH1 staining, we were only able to stain colon tumors from 28 patients in our study using IHC vs. 83 patients in TCGA, providing more statistical power to the TCGA data set. Furthermore, besides difference in the number of patients, different methods were used; IHC in our study vs. mass spectrometry in TCGA. Based on all of these factors, it is clear that additional studies should be done to assess the functional relationship of MSI2 and CDH1 expression. This point was added to the Discussion section.

**Point #2**. The whole idea of this manuscript to show the significance of MSI2 in prognosis colon cancer progression. It would be better to use regression and ROC analysis methods to show the prognostic significance of MSI2 in predicting disease progression.

**Response #2**: Thank you for your comment. We applied Cox regression analysis and ROC analysis to the data; however, these methods sdid not show any significance in relationship of MSI2 expression in primary CRC and prognosis in patients. We added this statement to the manuscript Results section.

**Point #3**. Figure 1A: MSI2 expression (representative IHC images) in adenocarcinoma and liver metastasis is not clear and hard to interpret. Higher magnification insert would help in compression between them.

**Response #3**: We apologize for the quality of the initially submitted images. We have updated Figure 1A, and replaced old images with the new images in this Figure.

**Point #4**. Figure 1D: Author showed MSI2 expression comparison between grade 3 with grade1 & 2. It would be better to show comparisons among normal and all the grades with representative IHC images of MSI2.

**Response #4**: We added new images with Grade 1 – 3 tumors (MSI2 IHC staining).

**Point #5**. Figure 4G-H: typo error in Y axis, % colony formation instead of % cell viability.

**Response #5**: We apologize for this typo. We updated the Y-axis in Figures 4G and 4H.

We once again thank the Editor and the Reviewers for a fair review, and hope that the improved manuscript would be suitable for PLoS One.

Sincerely yours,

Yanis Boumber, MD, PhD

Division of Hematology / Oncology

Section of Thoracic / Head Neck Medical Oncology

Robert H Lurie Comprehensive Cancer Center

Feinberg School of Medicine

Northwestern University, Chicago, IL, USA

Office: 303 E. Superior St, Room 3-111,

Phone: 312-503-4448

Email: [yanis.boumber@northwestern.edu](mailto:yanis.boumber@northwestern.edu)

Leonid Kharin, MD

Molecular Therapeutics Program

Fox Chase Cancer Center, W406,

333 Cottman Avenue

Philadelphia, PA, 19111, USA *and*

Thoracic-Abdominal Department

National Medical Research Centre for Oncology

14-liniya st. 63, 344029, Rostov-on-Don, Russia

e-mail: kharinleonid@gmail.com
